# Advancing Edge Speeds of Epithelial Monolayers Depend on Their Initial Confining Geometry

**DOI:** 10.1371/journal.pone.0153471

**Published:** 2016-04-14

**Authors:** Somanna A. Kollimada, Ankur H. Kulkarni, Aniket Ravan, Namrata Gundiah

**Affiliations:** 1 Department of Mechanical Engineering, Indian Institute of Science, Bangalore, India; 2 Department of Molecular Reproduction, Development and Genetics, Indian Institute of Science, Bangalore, India; Emory University School of Medicine, UNITED STATES

## Abstract

Collective cell migrations are essential in several physiological processes and are driven by both chemical and mechanical cues. The roles of substrate stiffness and confinement on collective migrations have been investigated in recent years, however few studies have addressed how geometric shapes influence collective cell migrations. Here, we address the hypothesis that the relative position of a cell within the confinement influences its motility. Monolayers of two types of epithelial cells—MCF7, a breast epithelial cancer cell line, and MDCK, a control epithelial cell line—were confined within circular, square, and cross-shaped stencils and their migration velocities were quantified upon release of the constraint using particle image velocimetry. The choice of stencil geometry allowed us to investigate individual cell motility within convex, straight and concave boundaries. Cells located in sharp, convex boundaries migrated at slower rates than those in concave or straight edges in both cell types. The overall cluster migration occurred in three phases: an initial linear increase with time, followed by a plateau region and a subsequent decrease in cluster speeds. An acto-myosin contractile ring, present in the MDCK but absent in MCF7 monolayer, was a prominent feature in the emergence of leader cells from the MDCK clusters which occurred every ~125 μm from the vertex of the cross. Further, coordinated cell movements displayed vorticity patterns in MDCK which were absent in MCF7 clusters. We also used cytoskeletal inhibitors to show the importance of acto-myosin bounding cables in collective migrations through translation of local movements to create long range coordinated movements and the creation of leader cells within ensembles. To our knowledge, this is the first demonstration of how bounding shapes influence long-term migratory behaviours of epithelial cell monolayers. These results are important for tissue engineering and may also enhance our understanding of cell movements during developmental patterning and cancer metastasis.

## Introduction

The motility of cells is essential in many physiological processes including developmental patterning which guides embryogenesis [1, 2], wound closure [3], immune response by white blood cells [4], and in the uncontrolled movement of metastatic cells in most cancers [5]. Whereas chemotactic stimuli are known to initiate and guide these responses, they alone are insufficient to explain the different rates of cell migrations, formation of tissues through morphogenesis, and the measured forces underlying these processes [1]. Several researches have emphasized the role of mechanical stimuli in collective cell migrations [6–8]. Studies also showed that geometric constraints dictate how mechanics affects cell growth and apoptosis [9]. Single adherent cells cultured on patterned shapes have cell-matrix adhesions that are mediated by traction forces which vary with surface convexity [10]. These studies suggest that stress fibers within the cell cytoskeleton reorganize to reduce membrane tension effects due to the cell boundary [11]. Periodic variations in the curvature of dynamically varying cell boundaries are associated with local cellular expansion and retraction which in turn drive individual cell motility [12, 13]. Geometric constraints hence influence the cell shape, force generation, and mechanisms for growth and development at the level of individual cells. The effects of geometry on cell clusters and their migration however remains poorly understood.

Recent studies show that cells confined within circular stencils rotate within the constrained geometry in a synchronized and coordinated manner [14]. Cell sheets expand non-uniformly through dynamic instabilities upon release of the constraint and develop finger-like projections along their boundaries which determine the overall monolayer migration directions [15, 16]. Cooperativity between individual cells is mediated *via* cell-cell cadherin connections which are crucial in transferring loads that direct and facilitate monolayer movements along lines of minimal intercellular shear stress [17]. Computational models exploring motility in dense cellular monolayers show that the size of cluster populations is an important parameter for maintaining coordinated movements during migration [18]. The presence of an acto-myosin ring, analogous to a purse-string contraction in wound healing, has been identified as being vital in defining the presence of leader cells in the advancing edge of the cluster [19]. A breakdown in the acto-myosin cables in regions of sharp convex curvatures correlates with the emergence of leader cells from cluster borders [20, 21]. Together, these studies demonstrate the importance of the cluster boundary to the overall migration behaviours.

Do the spatial positions of cells within a confined geometry dictate the rate and direction of its movement relative to its neighbours? To answer this question, it is necessary to characterize the cytoskeletal organizations of cell clusters due to mechanical constraints and quantify their migration velocities over time. We selected MCF7 and MDCK cells based on differential epithelial-like character and mechanically confined them in circle, square, and cross shapes. Both cell types are of epithelial origin and have been used in earlier studies on collective cell migrations; the MCF7 cells do not however show coordinated rotations in contrast to those reported by MDCK cells [14]. The choice of the two epithelial cell lines was also motivated with an aim of exploring differences in the behaviors of fast moving MDCK cells with those of cancer cells (MCF7); both cells however express cadherin which is crucial in the transfer of loads during collective cell migrations. We measured differences in the spatio-temporal motility of cells in regions with convex, straight and concave geometries upon release from the constraint to explore the effects of geometric curvatures on collective cell migrations. Because the cross shape in our study also included straight sections along the arms, convex regions at the vertices, and concave regions at the intersections of the two arms, we compared the migration velocities of MCF7 and MDCK cells initially constrained in the cross shapes which have not been reported earlier. Our studies establish that the motility of individual cells in the monolayer cluster is a function of their specific location within the confined geometry. These results are important for tissue engineering and may also enhance our understanding of cell movements during developmental patterning and cancer metastasis.

## Materials and Methods

### Cell Culture

We used MCF7 breast cancer cells and MDCK epithelial cells to compare differences in collective migration responses due to the constraining effects of bounding shapes in our experiments. The cell lines were separately grown in T25 flasks in a medium of high glucose DMEM solution containing L-glutamine and supplemented with 10% fetal bovine serum in an incubator maintained at 37°C and 5% CO_2_. Cells were trypsinized, seeded on collagen I coated plastic petridishes, and grown to confluence within the constrained shape as described below. We explored the individual contributions of myosin-2 and actin in the overall cellular migration speeds and behaviours using inhibitors. To disrupt myosin-2 activity on MCF7 and MDCK cells grown in the constrained cross geometries, we used blebbistatin (50 μM, Sigma-Aldrich, India). We also used cytochalasin-D (2 μM) to depolymerize actin networks in the cell clusters in the migration studies.

### Fabrication of stencil used as geometric constraint

**Fig 1:**
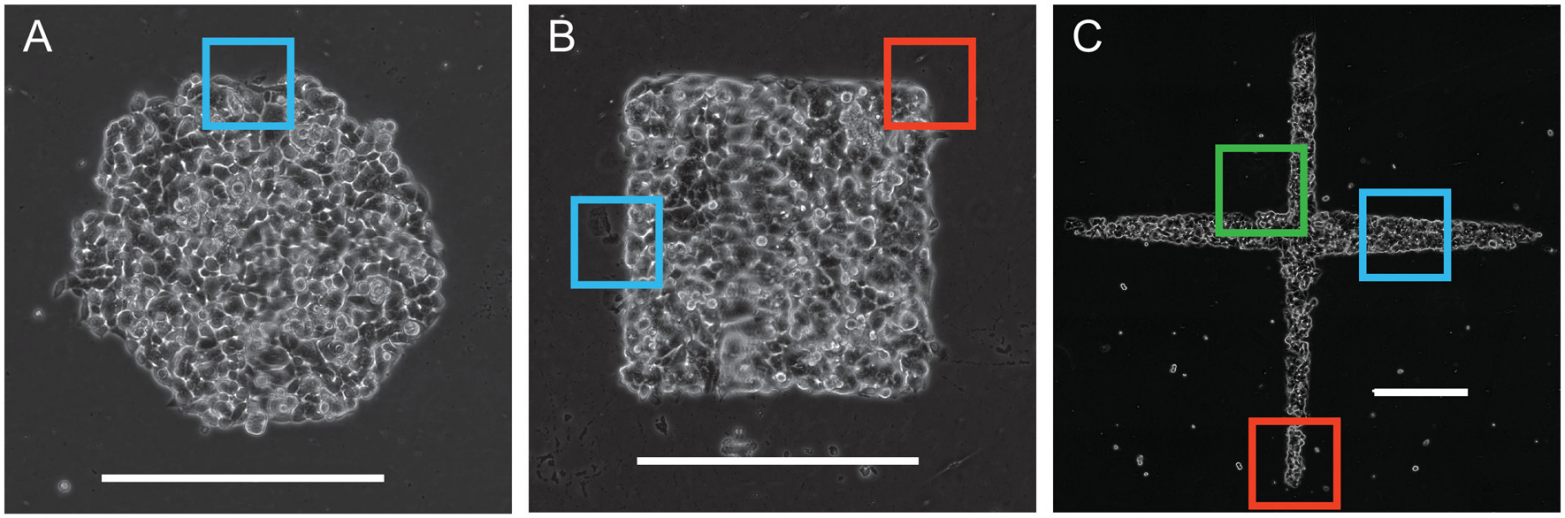
MCF7 cells at the time of release from circle (A), square (B) and cross (C) shaped constraints are shown. A representation of the regions chosen as the vertices (red), sides (blue) and intersections (green) in the three shapes are indicated in the boxes. Scale bar = 500 μm.

We fabricated PDMS stencils using a replica molding technique which began with stainless steel stencils with the imprints of circle, square, and cross shaped geometries of ~25,000 μm^2^ area (Fig 1). Poly dimethyl siloxane (10:1 base monomer-to-crosslinker ratio, PDMS; Sylgard^®^ 184, Dow Corning) was poured on the metal stencil to prepare a negative imprint, the PDMS stencil treated with tri-chloro di-methyl silane to prevent adhesion, and a second layer of PDMS poured with a silanized glass slide pressed to remove excess material. PDMS was cured and peeled to obtain a soft sheet replica of the metal stencil was used in the study. A single cross, circle, and square pattern, each separated by a distance of ~500 μm from the next shape, was present on each sheet and was designed to avoid interactions between the cluster fronts during migration. The sheet was again silanized, to prevent cell adhesion, sterilized, and pressed on a collagen coated petridish to permit cell attachment only in the geometric patterns. The entire assembly was sterilized using UV for 30 minutes. MCF7 or MDCK cells were seeded within constraints, cultured to confluence, and the PDMS stencil gently peeled off from the petridish surface to remove the constraint from the patterned clusters for migration studies. A mosaic image of the cell cluster was obtained using an inverted microscope (Leica DMI6000) equipped with a live cell stage and a 10X phase contrast objective. Images were obtained every 10 minutes to characterize the cluster migrations over 48 hours for MCF7 and 16 hours for MDCK cells to characterize the temporal variations in cell migrations. At the end of migration, cells were fixed in 4% paraformaldehyde/ PBS solution, permeabilized using 0.5% Triton X-100 in PBS, and treated with Rhodamine Phalloidin (Molecular Probes; R415) to visualize actin fibers and DAPI (Molecular Probes; D1306) to stain the nuclei. Additional samples were fixed in PBS containing 4% paraformaldehyde (w/v), permeabilized with 0.1% Triton-X 100 (Sigma) (v/v) in PBS for 10 minutes, blocked with 3% BSA (Sigma) for 60 min at room temperature and incubated with primary anti E-Cadherin antibody (1:200, CST) overnight at 4°C. The sample was washed thrice with PBS and incubated using secondary Alexa-Fluor 488 conjugated anti-rabbit antibody (1:400, Cell Sign. Tech) for 60 min at room temperature to visualize E-Cadherin and the sample counterstained using DAPI (1:400, Molecular Probes). Samples were imaged with a confocal microscope (Leica Microsystems, TCS SP5 II) using 10X, 40X and 63X oil immersion objectives.

### Image Analysis

We quantified cell motility using Particle Image Velocimetry (PIV) using cross-correlations of sequential spatially-calibrated images to obtain the velocity fields of the migrating cell clusters (MatPIV 1.6.1). Vorticities were derived as curl of the measured velocity fields. Using these methods, we calculated the velocities corresponding to cells located in the advancing edges of sides, vertices and intersections in the circle, square and cross shapes by defining a small region of interest corresponding to the specific locus of points (Fig 1). In addition, we tracked the centroids of individual cells using an automated custom written MATLAB code. Briefly, we converted the phase contrast image of the cell within a circular region of interest to a grayscale one and determined the centroid based on the image intensity. We used this cell centroid position to define a new region of interest in the corresponding image which enclosed the migrating cell. This iterative process hence helped locate the new centroid locus and permitted us to automate the detection process for many cells (S1 Video). We tracked a total of 20–43 cells in each of the different regions in the circle, square and cross shapes respectively.

### Statistical Analysis

We analysed statistical differences using ANOVA with Bonferroni comparisons to test for regional variations in the cell speeds within each shape. All data are reported as average ± standard deviations in each experiment. A p ≤ 0.05 was considered statistically significant.

## Results and Discussion

### Migration velocities depend on the geometry of the confining shape and evolve in three phases

**Fig 2:**
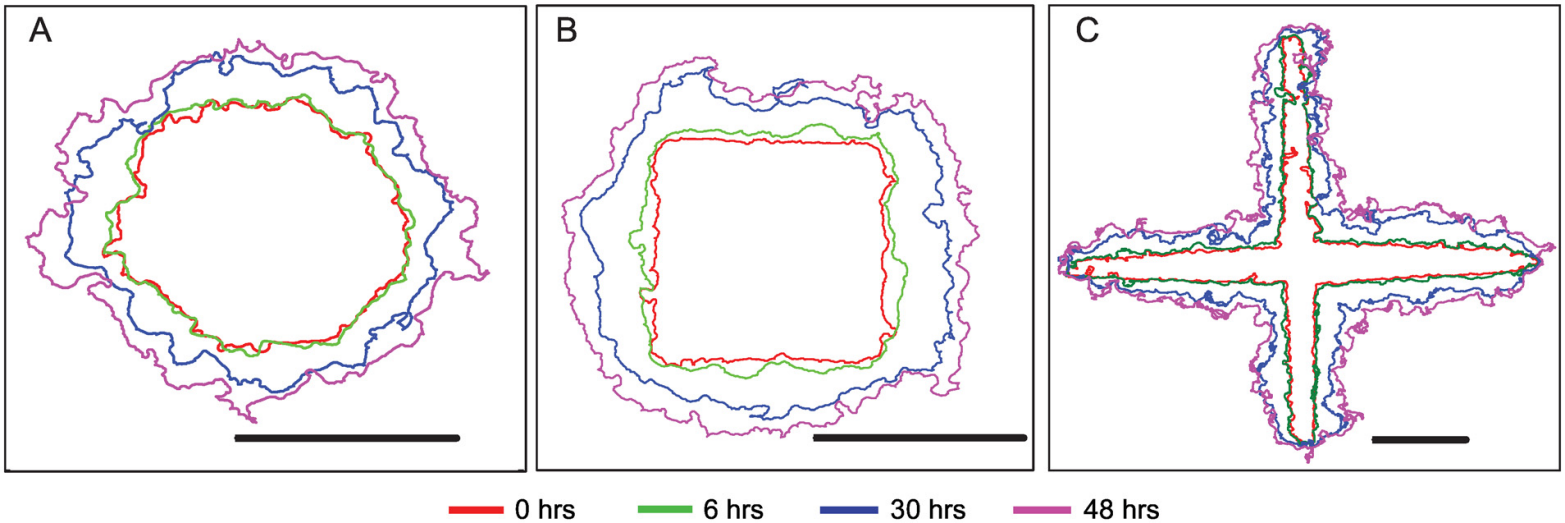
The edges of the MCF7 clusters in circle (A), square (B) and cross (C) are shown at the start of the experiment (red), at 6 hours (green), 30 hours (blue) and 48 hours after removal of constraint (magenta). Scale bar = 500 μm.

Fig 2 shows outward movements of the MCF7 monolayer boundary over 48 hours after removal of the bounding constraint to explore temporal variations in the cell migrations. In addition, we see spatial variations in the migration behaviours of the monolayers which were initially constrained in the circle (S2 Video), square vertex (S3 Video), cross intersection (S4 Video) and cross vertex (S5 Video) geometries. In all cases, we see minimal movement in the MCF7 cell clusters at the end of 6 hours upon release of the constraint following which the cell boundaries evolved from a relatively smooth edge to that with high roughness. The edges smoothened at the sharp convex vertex regions of the square by about 30 hours (Fig 2B). In contrast, we see minimal movements in cells located at the vertices of the cross as compared to the corresponding edges and intersections (Fig 2C).

**Fig 3:**
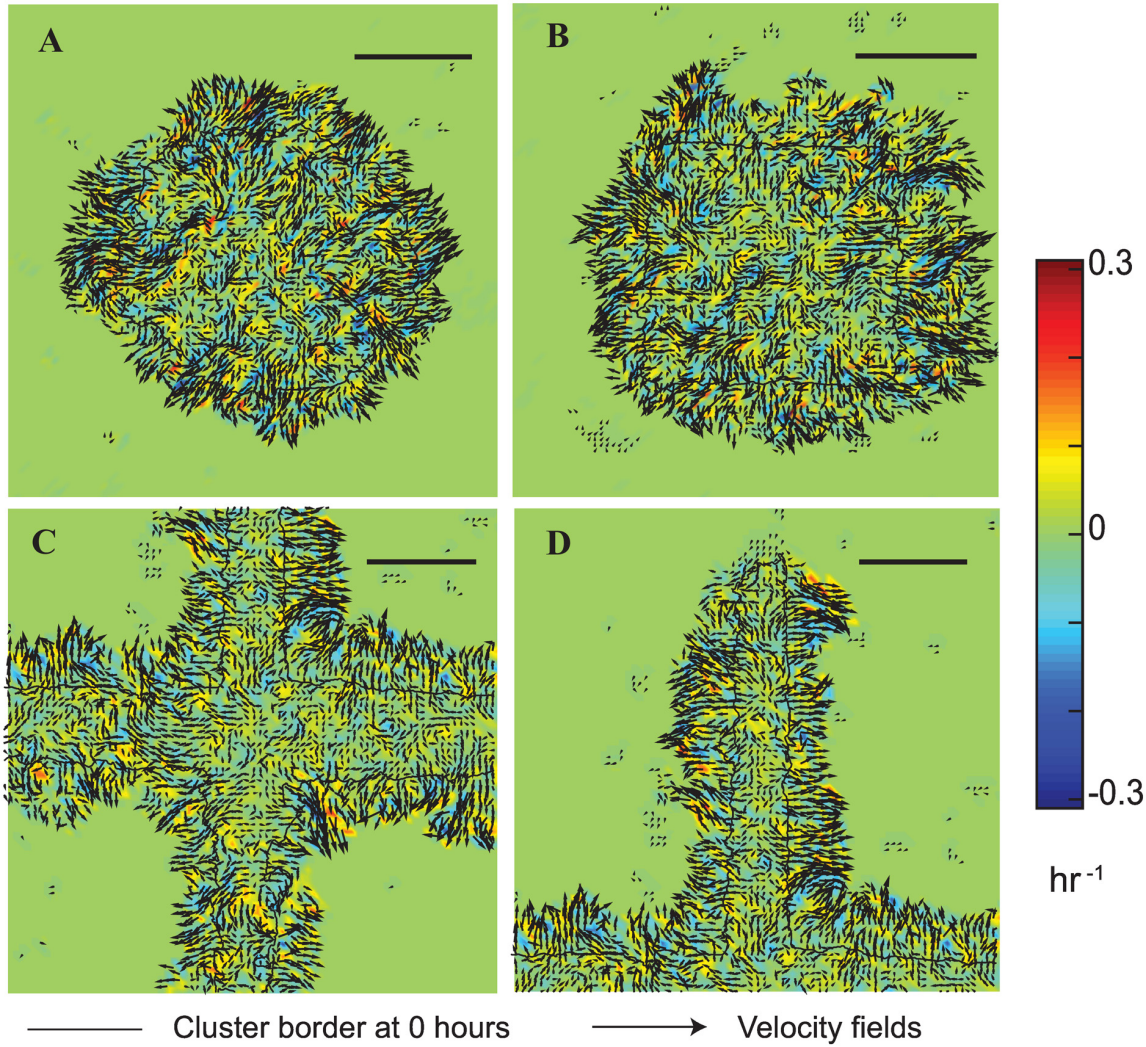
Velocity and vorticity fields in MCF7 cell monolayers in the circle (A), square (B), cross vertex (C) and intersections (D) at 30 hours summed every hour. The solid black line is the cluster border at the start of migration. Scale bar = 250 μm.

Does the shape of the bounding region influence the migration velocities of the monolayer? To address this question, we quantified the velocity fields of initially constrained MCF7 cells using PIV for the entire circle (Fig 3A) and square shapes (Fig 3B), summed every 6 hour over the entire 48 hour duration, and shown at 30 hours in Fig 3. Corresponding regions from the cross vertex (Fig 3C) and intersections (Fig 3D) are also shown at 30 hours. These results show that the velocities are highest at the cluster edges as compared to the monolayer interiors. Corners of the square and the vertices of the cross had less motility as compared to other regions. These data also show large outward diagonal movements in the cell monolayer at the intersections of the cross (Fig 3D). To investigate spatial differences in velocities for the three shapes, we defined a 100 μm region at the cluster tips as the vertex region for the square and cross shapes whereas we used a 200 μm region to represent the cross intersection. We summed data from 6 hour period which show three distinct phases in the migration speeds of the MCF7 clusters. Cells in the circle moved out radially with an initial speed of 1.57±0.26 μm/hr which increased linearly to 3.83±0.51 μm/hr by 18 hours and finally reduced to 2.58±0.30 μm/hr at 42 hours.

**Fig 4:**
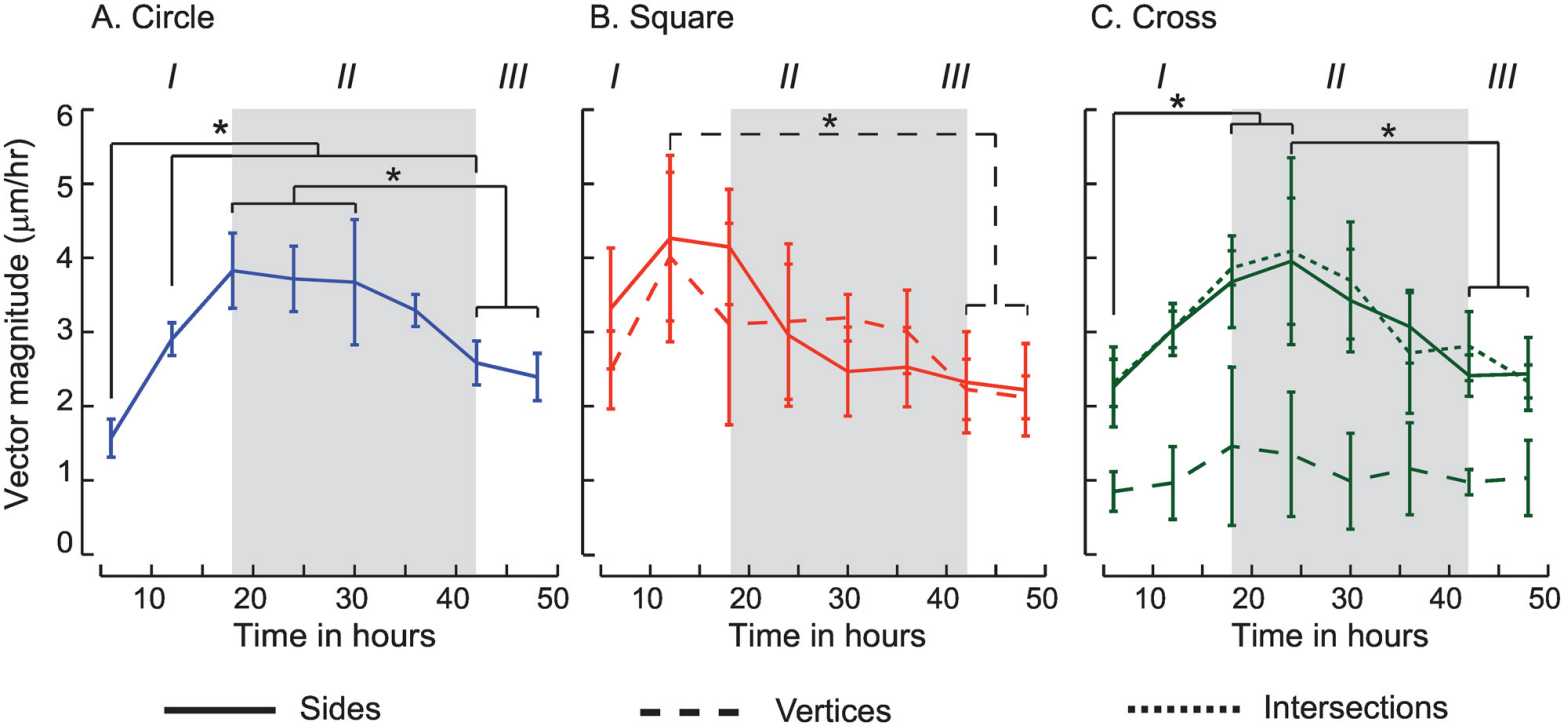
Cluster speeds show temporal differences in the different regions of the circle (A), square (B) and cross (C) shapes respectively. Speeds from the sides increase (I), plateau (II) and finally decrease (III) over time. These results show clear differences in cluster edge speeds which depend on spatial location in the geometry. Significant differences (p<0.05) in speeds are indicated in each figure.

We explored the spatio-temporal dependence of migration speeds in the square and cross monolayers which include a region of convex curvature at the vertex of the square and cross, and those with concave curvatures at the cross intersections. These results show a similar temporal variation in the migration speeds which may be delineated into three regions (Fig 4). We do not see any differences in the speeds of cells from the vertex regions of the square with the corresponding sides. In contrast, the speeds from cells in the vertex of the cross were 64% lower than those in the corresponding intersections and sides and also show relatively minimal movement of vertices over the 48 hour duration. Together, these results clearly show that migration behaviors of cell clusters have a functional dependence on their spatial location within the cluster shape.

**Fig 5:**
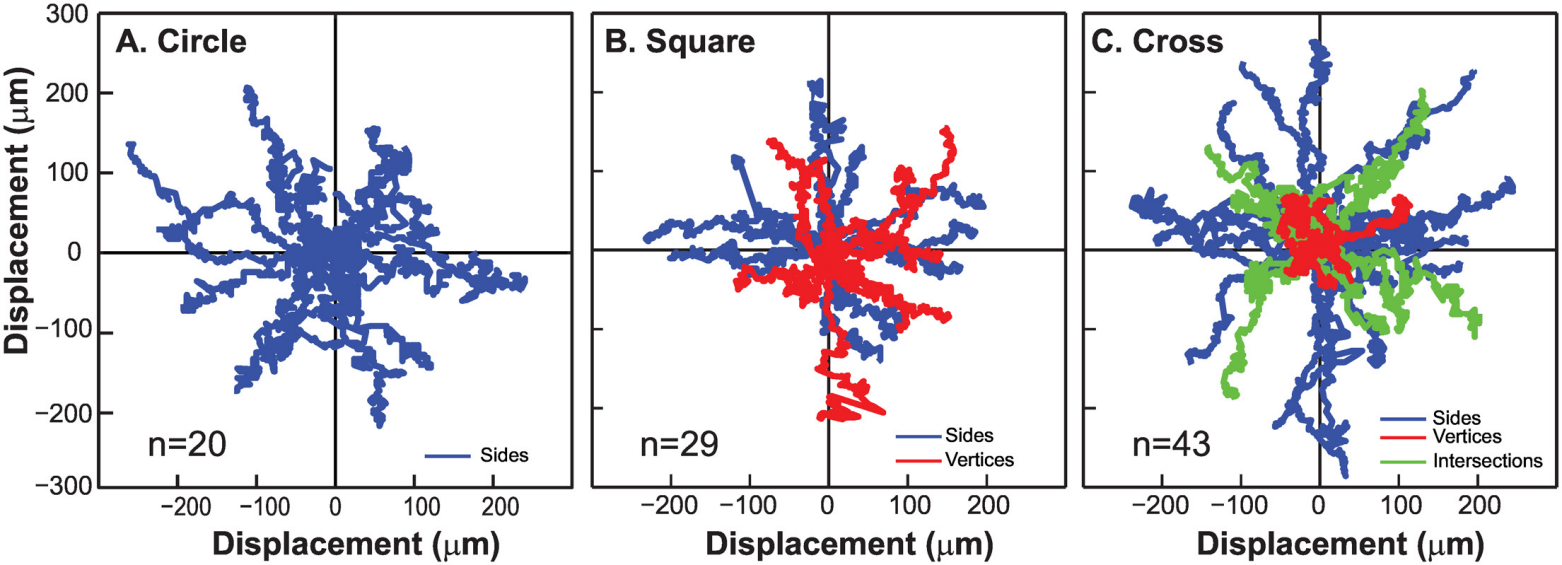
Migrations of MCF7 cells located on the advancing edges of the circle (A), square (B) and cross shapes (C) were obtained using individual cell tracking and are illustrated on a windrose plot to show the cell migration trajectories from the cluster edge. We see differences in motility that depend on the cell spatial positions which are labelled corresponding to sides, vertices and intersections.

To quantify the migrations of individual cells from each side, vertex (square and cross shapes) and intersection (cross alone), we tracked cells and plotted the results (Fig 5) over a 48 hour period. These results show that cells in the sides (blue) for each of the three shapes move outwards normal to the boundary, although there were no differences in cell speeds between the three shapes respectively. In contrast, cells at the cross intersections (green) move diagonally outward and have similar displacements as those located in the sides of the square. Cells at the cross vertices (red) however show relatively small displacements. Because the cross shape had a very sharp curvature at the vertex regions as compared to cells located in the square vertex, our results show that sharp convex shapes alone influence cell migrations.

**Fig 6:**
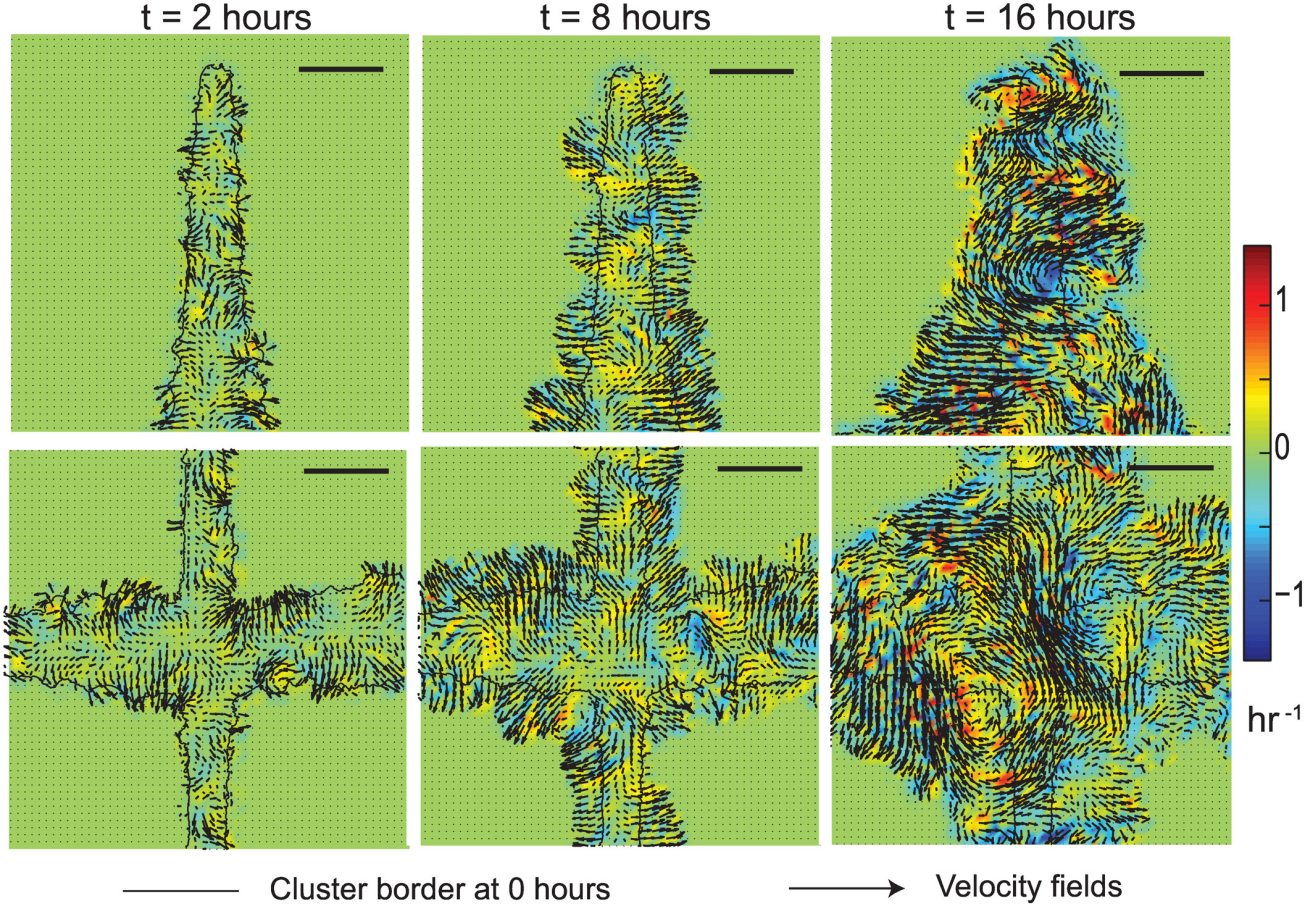
Velocity fields and vorticity maps for MDCK cells are shown at 2 hours (A, D), 8 hours (B, E) and 16 hours (C, F) after removal of constraint. The cluster border is also shown at 0 hours for reference. Cells in the vertices show minimal movement as compared to those in the sides and intersections (D, E, F). We see the development of vortices, in both clockwise (blue) and counter clockwise (yellow-red) directions at intersections by 16 hours. Scale bar = 250 μm.

In contrast to the MCF7 cell migrations in the cross shape, MDCK cells uniformly spread out within the cross shape upon removal of confinement (S6 and S7 Videos). Upon release from the constraint, we see a clear directionality in the cell migration behaviors. MDCK cells initially move normal to the surface and the individual cells align with their short axis normal to the direction of migration (Fig 6B and 6E). Following this, we start seeing the presence of synchronized, coordinated rotations by 8 hours at the intersection regions of the cross and in the arms by 16 hours (Fig 6C and 6F). Vortices were however absent in MCF7 clusters following removal of the constraint (Fig 3, S4 and S5 Videos).

**Fig 7:**
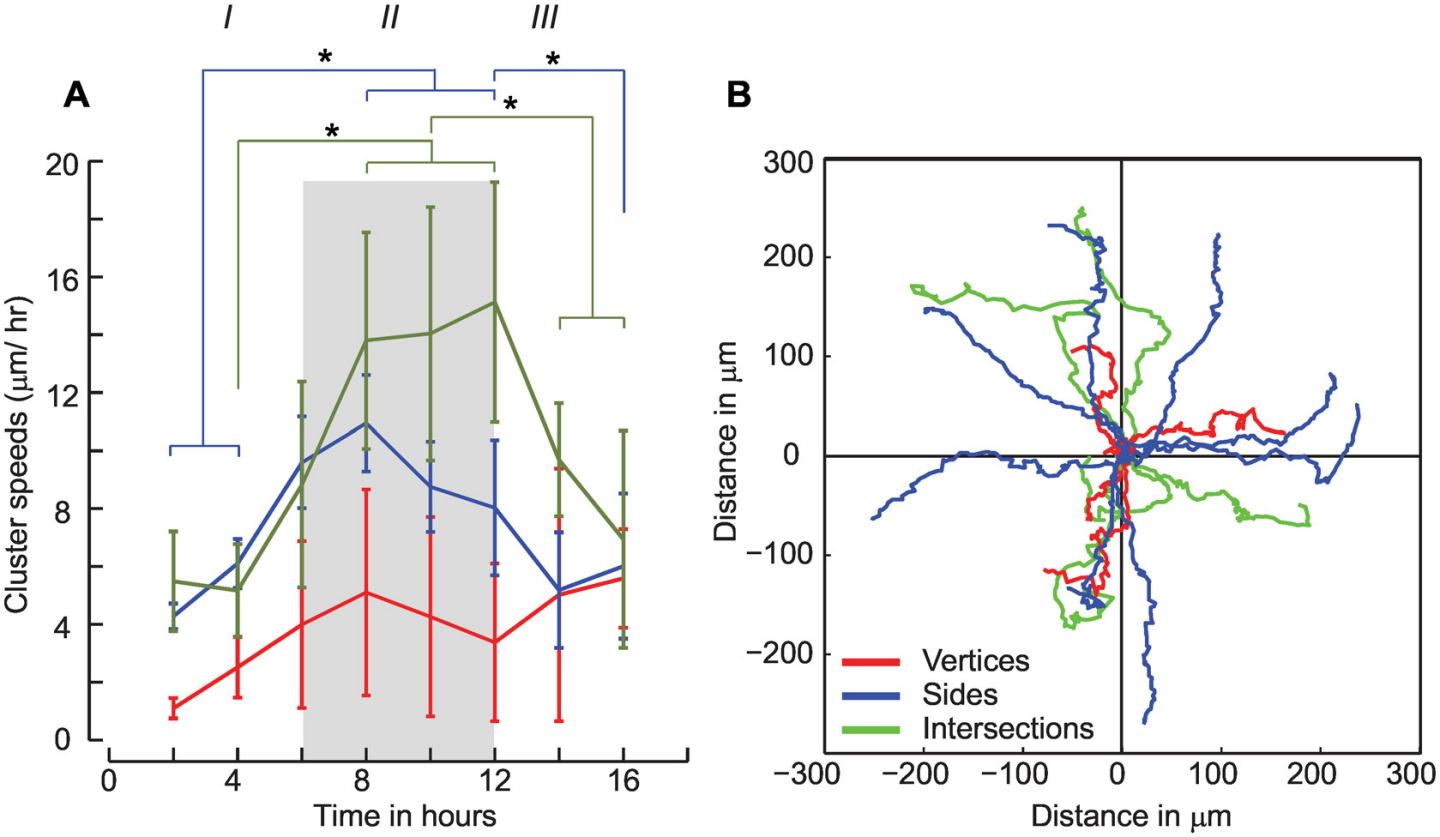
A: We delineate three phases in the MDCK clusters based on the temporal speed variations of initially constrained cells in the cross shape in regions corresponding to sides, vertices and intersections (p<0.05). We see an increase in the speeds of cells from the cluster sides and vertices up to 8 hours (Phase I), a plateau region observed until 12 hours (Phase II), followed by a decrease in the cluster speeds by 16 hours (Phase III) B. Cell trajectories depend on their spatial location in the cross shape.

We used the PIV results to quantify spatial differences in cell speeds after removal of the cross constraint (Fig 7). We defined a 200 μm region corresponding to the intersections of the cross and 80 μm regions to delineate vertices of the cross. These results show a temporal dependence on the speeds of the MDCK cells which are similar to that observed for MCF7 cells. Results show a linear increase in the speeds of cells located in the sides of the cross from initial values of 4.27±0.44 μm/hr at the time of release from the constraint. Phase II is characterized by a plateau region of constant cell speed of 10.94±1.66 μm/hr at 8 hours which decreases to 6.01±2.51 μm/hr (Phase III) at the end of 16 hours. Cells located at vertices have significantly lower migration speeds at 2 hours following release of the constraint (1.09±0.35 μm/hr) which increased to 5.59±1.71 μm/hr at 16 hours. These trends in the spatio-temporal migration speeds are similar to those for the MCF7 clusters (Fig 4). However, migration speeds for the MDCK cells were higher (4.27±0.44 μm/hr) in regions corresponding to the sides of the cross at 2 hours as compared to corresponding regions in the MCF7 clusters at 6 hours (2.12±0.55 μm/hr).

Mechanical mechanisms, hypothesized to drive wound healing, are characterized by cell crawling that is generally accompanied with force polarization and cell spreading on the substrate, cell-cell adhesions which are crucial to keeping the monolayer cohesive, and cell proliferations [22]. Results from our study clearly show the importance of cell spreading on the cluster expansions (S6 and S7 Videos). Cells in the MDCK clusters are well spread and have the highest speeds by 10 hours followed by a decrease in the cell speeds (Fig 7A). Computational models which quantify changes in the cluster borders with time show that the average velocity increases proportional to the time since wounding [23]. Previous studies also show that MDCK cells exhibit contact inhibition and do not begin dividing until the spread cell areas reach a threshold value [24]. In contrast, MCF7 cells continue to divide after confluence [25] and also show a linear dependence of the cluster speeds with time for about 20 hours which is also accompanied with a subsequent decrease in the cluster speeds (Fig 4). Experimental data from our study confirm the findings related to cell speed increase and demonstrate the importance of cell spreading to the overall migration speeds. To date, few models or experimental studies have shown the plateau in the cluster speeds and the corresponding decrease which accompany cell spreading and proliferations. Additional studies are clearly warranted to explore the individual effects of proliferation and cell spreading proposed in current computational models of collective cell migrations.

### Motion of epithelial cells and the creation of leader cells in an advancing front

**Fig 8:**
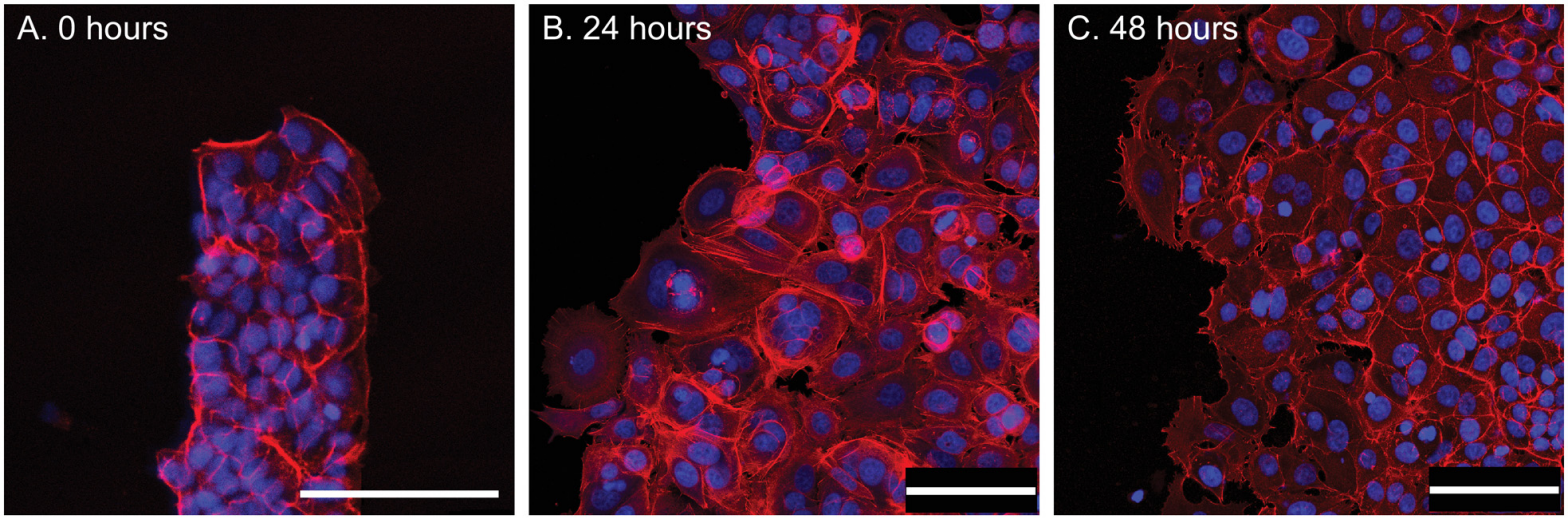
Fluorescence images show actin (red) and the nucleus (blue) for MCF7 clusters at 0 hours (A), 24 hours (B), and 48 hours (C) after release from constraints. There is no clear cable-like structure bounding the cluster. Scale bar = 100 μm.

Actin cytoskeletal networks get recruited at the leading edges of cells which polarize and permit force generations that are essential in migrations. We hypothesized that the actin cytoskeletal networks have a preferential organization which are a function of the shape of the constraining geometry. The presence and importance of an acto-myosin cable to the overall migrations has been shown earlier using MDCK cells [19, 20]. To visualize differences in actin distributions in the differing regions of the constraining geometry, we stained the MCF7 cell clusters to visualize the presence of nuclei and actin after the onset of migration (Fig 8). These results show a high cell density in the constraining shape at the time of release of the constraint. However, we do not see any preferential organization of actin at the boundaries of the constraining stencil at the start of the experiment, at maximum cluster speeds (24 hours) and during deceleration in the cell migrations at 48 hours. Cells spread over from the monolayer borders over time and migrate via lamellipodia but maintain the sheet integrity over time.

**Fig 9:**
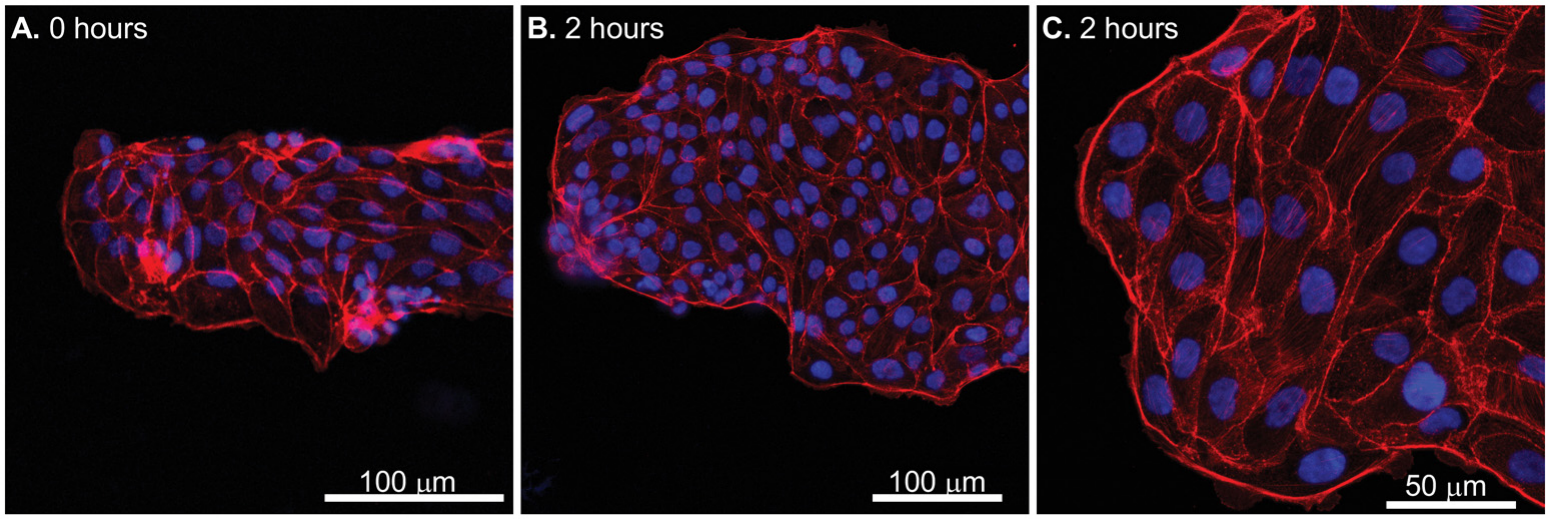
MDCK cells were stained for actin (red) and nucleus (blue) and are shown at 0 (A) and 2 hours (B) after removal of constraint. The acto-myosin cable at cluster borders is visible. Higher magnification (C) image clearly shows the presence of the actin cable.

To compare these results with MDCK cell clusters, we visualized the clusters at different times during migration and clearly show the presence of bounding acto-myosin cables (Fig 9) 2 hours after removal of the geometric constraint which persist throughout the experiment in contrast to results from the MCF7 cluster migrations where the acto-myosin cable is absent throughout the duration of the experiment (Fig 8). Recent work from Reffay and coworkers also show the presence of similar cables in MDCK clusters. Further, using laser ablation they showed that the cable is maintained under tension [26]. Studies also suggest that the cable activates RhoA signalling through mechanical tension and prevents the initiation of new leader cells in the finger-like projections [26]. Cells in the monolayer clusters orient and migrate along directions of minimal intercellular shear in a wave-like pattern which coincides with the propagation of MAP kinase in the monolayer [15, 22]. Earlier studies used traction force measurements to show the propagation of a mechanical wave which spreads across the whole sheet [19]. This leads to heterogeneous stress distributions and also serves as a mechanism for long range guidance and pattern formation in epithelial cell clusters [17]. Our results show directional heterogeneity in cell motility that is dependent on the boundary shape (Fig 7A) which may also be linked to the acto-myosin cable localized to the advancing front. Although this directional heterogeneity in migration speeds based on the boundary shape was present, vorticity patterns and the acto-myosin cable were both absent in the MCF7 cell migrations in our study.

**Fig 10:**
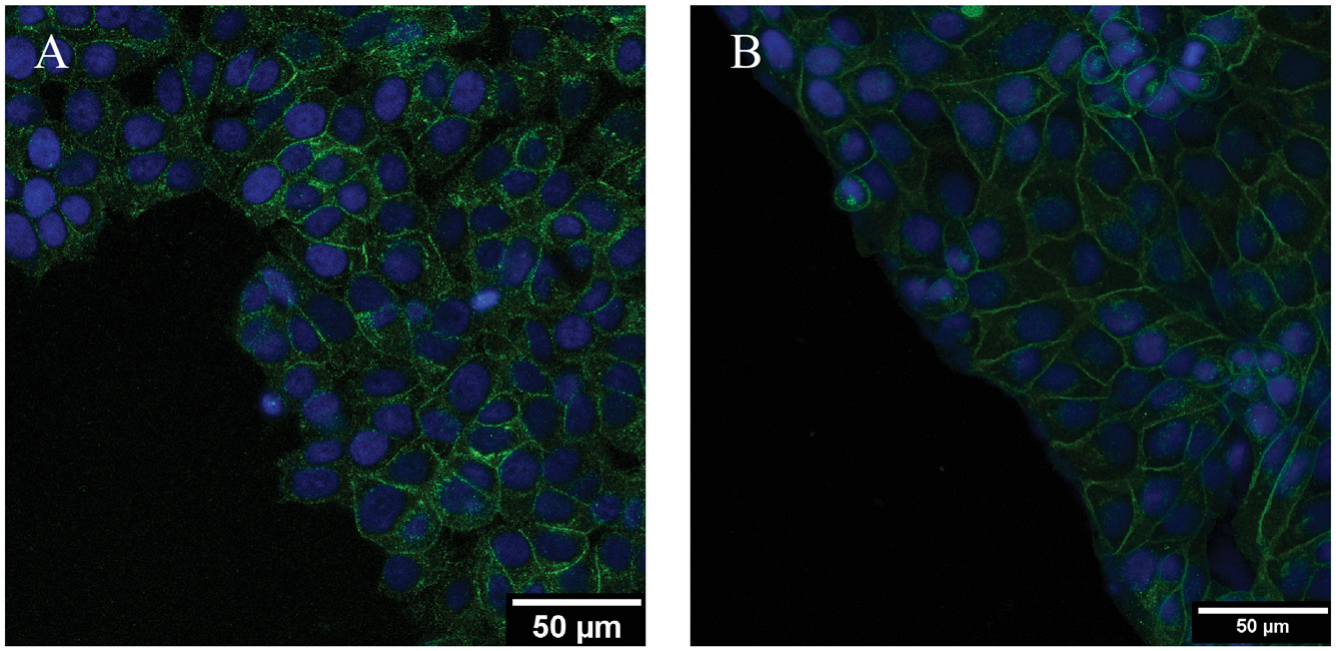
Clusters were stained to explore the presence of E-cadherin in A. MCF7 clusters and B. MDCK cells constrained in the cross shape. The cell nucleus was labelled using DAPI.

Synchronized collective rotations in cell monolayers and three dimensional constructs are possible through transfer of forces *via* E-cadherin regulated intercellular cell adhesion [12]. Although MCF7 cells express E-cadherin when cultured in monolayers (Fig 10A), we do not however see a significant presence of vortices even at 30 hours after removal of the constraint (Fig 3). The presence of E-cadherin is clearly visible in the MDCK clusters (Fig 10B); swirling vortices have been reported in MDCK cells but are absent in breast cancer epithelial cells which were retained within constrained circular patterns [14]. These results suggest that although E-cadherin is essential in force transfer and in maintaining cluster integrity [14, 21–22], its presence alone does not result in the presence of vortices.

**Fig 11:**
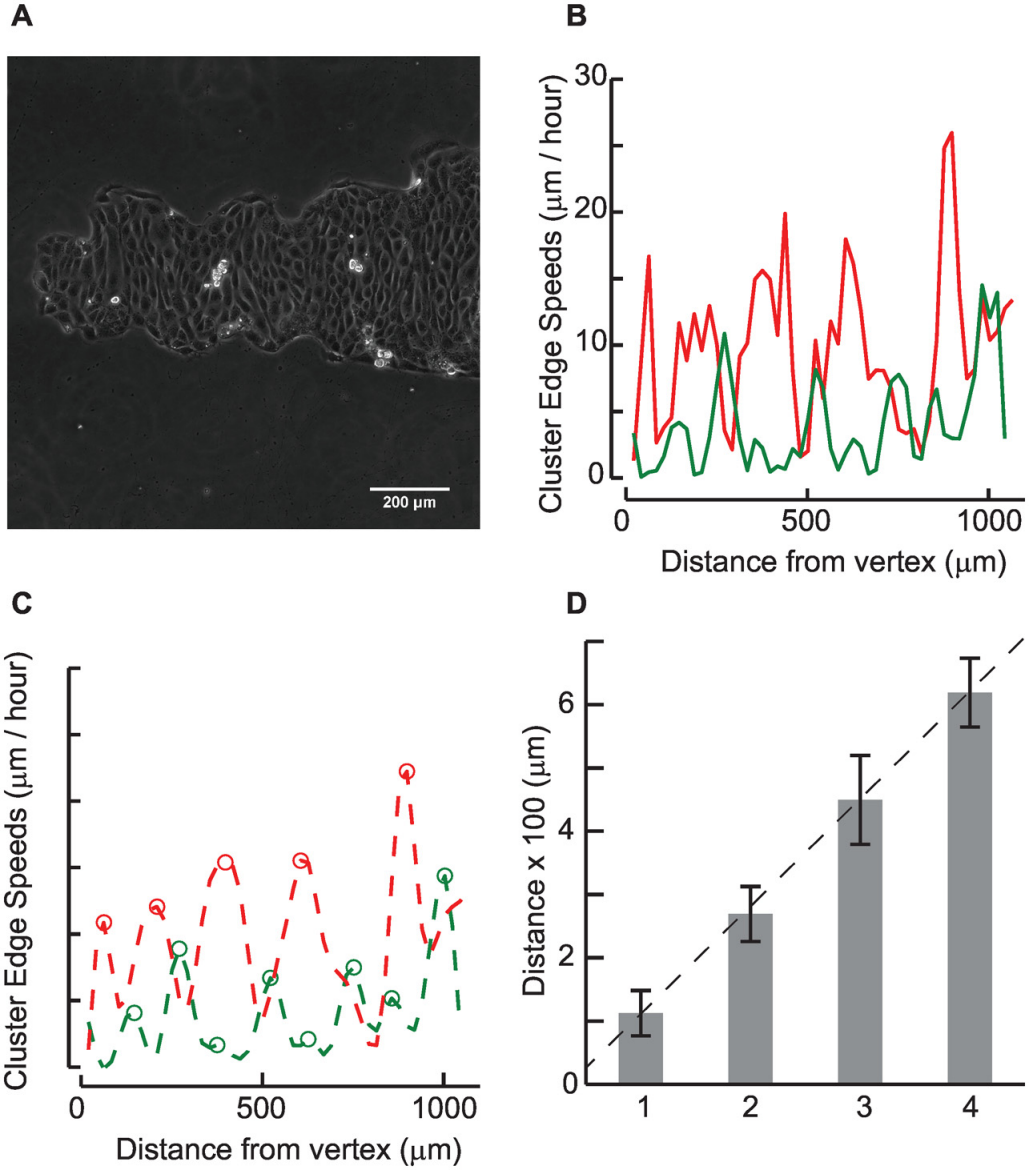
A. To assess emergence of leader cells in the MDCK clusters, speeds corresponding to the edges were quantified starting at the vertex marked using an asterisk in the figure. B. Raw data from MDCK cell clusters in cross shapes from two opposite edges at 2 hours after removal of constraint show variations in their speeds with location. C. Data were smoothened and the peaks were identified. D. Data were averaged from four cross arms to quantify average distances between peaks.

We explored possible periodicities in the emergence of leader-like cells which drag the cluster boundary outward using the PIV results from MDCK cell migrations anchored at the cross vertices. The vector magnitudes of boundary cells from the four arms of the cross were used to determine peaks in the speeds. The distance of the peaks from the vertex, on each alternating side of the four arms was determined after smoothening the raw data at 2 hours. Fig 11A shows an image of the MDCK cross arm and corresponding raw data for migration speeds of the advancing cluster front for this region where the wave like pattern is clearly visible (Fig 11B). To effectively detect the peaks in these date, we smoothed the migration speeds using a Butterworth filter with order 3 and a cutoff of 0.4. These parameters were optimized to smooth the local maxima which correspond to data from a single leader cell observed in each major peak. These data were used to determine the distance of the peaks from the vertex of the cross where the MDCK cells are relatively static (Fig 11C). We combined the results from all four arms (Fig 11D) that show the appearance of peaks at regular intervals of 125 μm starting from the cross vertex. Numerical models of one dimensional stretched membrane enclosing an active viscous fluid have been used to provide insights into the migration behaviors of epithelial cells [15]. Such models predict the appearance of finger-like structures at regular intervals of ~180 μm upon removal of geometric constraints which subsequently undergo branching. These formations emerge upon removal of geometric constraints and result in outward migrations of the MDCK clusters through the development of polarized and motile leader cells [15]. The tip cell, serving as a leader, is polarized from the front to the rear and these leader cells in the advancing front are functionally different from other cells comprising the cluster [27]. Ablation of the leader cells transiently perturbs cell cluster migrations which may interchange the individual roles of the leader and follower cells comprising the monolayer [28]. Transmembrane DDR1 protein locations reduce the myosin dependent contractility at cell-cell junctions through Par3 and Par6 polarity proteins and p190ARhoGAP [27]. Manipulation of Rac activity may also induce dominant leader cell-like behaviour [29].

### Importance of acto-myosin in collective cell migrations

To explore mechanistic effects on collective cell migrations, researchers have modelled cell monolayers as a heterogeneous elastic membrane sliding over a viscous sheet [30], visco-elastic material bounded by an elastic membrane [15], a collection of stochastic particles [31]. These models were mainly based on the migration response of individual cells comprising the monolayer with added cell-cell interactions to capture the overall monolayer dynamics. Lee and Wolgemuth based their model on these parameters and showed that wounds can close without the presence of an acto-myosin cable and exhibit the presence of finger-like projections [23]. Because all the cells were identical in the monolayer, the fingering instability was however not as pronounced as those seen experimentally [21]. In contrast, Mark and coworkers included the presence of an acto-myosin cable through tension in the membrane bounding the cluster monolayer and showed a clear fingering instability in their model [15]. Cells in the interior of the clusters are however under internal stresses due to confinement which was modelled through a pre-stretch in the monolayer using a discretized elastic membrane based model [30]. Collective cell motion in epithelial sheets, modelled as a group of stochastically interacting particles, also show the complex velocity field patterns and the emergence of leader cells which are observed in migrating MDCK cells. To explore the mechanistic principles that direct collective epithelial cell migrations, Tarle *et al* modeled the forces underlying the interactions between cell-cell neighbors, the orientation ordering of cells, and the tension due to presence of an acto-myosin cable [32]. Based on these interactions, they explored the main factors influencing the creation of leader cells and those involved in directing the cohort of cells behind the outward cluster front. The model also predicted an increase in finger formation and slower boundary expansion with inhibition of acto-myosin. The blebbistatin treatment in our study, aimed at inhibiting the formation of the acto-myosin cable, led to a dramatic decrease in border migration speed which is also predicted by Tarle and coworkers. Blebbistatin treatment causes a loss in the ability of the cells to generate contractile forces which are important in migration. We did not however see any additional instabilities (S9 Video), associated with the presence of leader cells, in our experiments using MDCK cells which were predicted by their model. In contrast, the instabilities are visible in the blebbistatin treated MCF-7 cell migrations (S5 and S8 Videos). Together, our studies using two different cell models show the combined effects of tension, generated via the acto-myosin cable, and cell contractility which control finger instabilities and drive cell migrations.

Rotations in the MDCK cell clusters are an intrinsic feature of epithelial cell clusters which are clearly absent in the MCF7 migrations in our study. Because these studies were performed on rigid substrates, the transmission of cell generated forces through the substrate is low. Studies by Tambe and coworkers also show that MCF10A cells, overexpressing markers which lead to decreased expression in cell-cell junctional proteins do not move along the direction of maximum principal stress in contrast to control MCF10A cells which express the same proteins. These studies show that cell-cell junctions are essential to most force transmissions during migrations. Further, we do not see a clear pattern for the emergence of leader cells from the MCF7 cell monolayers that also did not exhibit any acto-myosin cables surrounding the clusters. Downregulation of adherens junctions is reminiscent of cancer cells and is associated with uncoordinated movements in constrained cell monolayers [14]. Our experiments included the MDCK cell type which had a prominent acto-myosin cable and the MCF7 which exhibits low collective behaviour [14] and did not have the bounding cable. The use of constraining geometries with differential curvatures in this study did not facilitate periodicity in the emergence of leader-like cells in the MCF7 cells but was accompanied with lower cluster speeds in regions of convex curvature which is not reported earlier.

**Fig 12:**
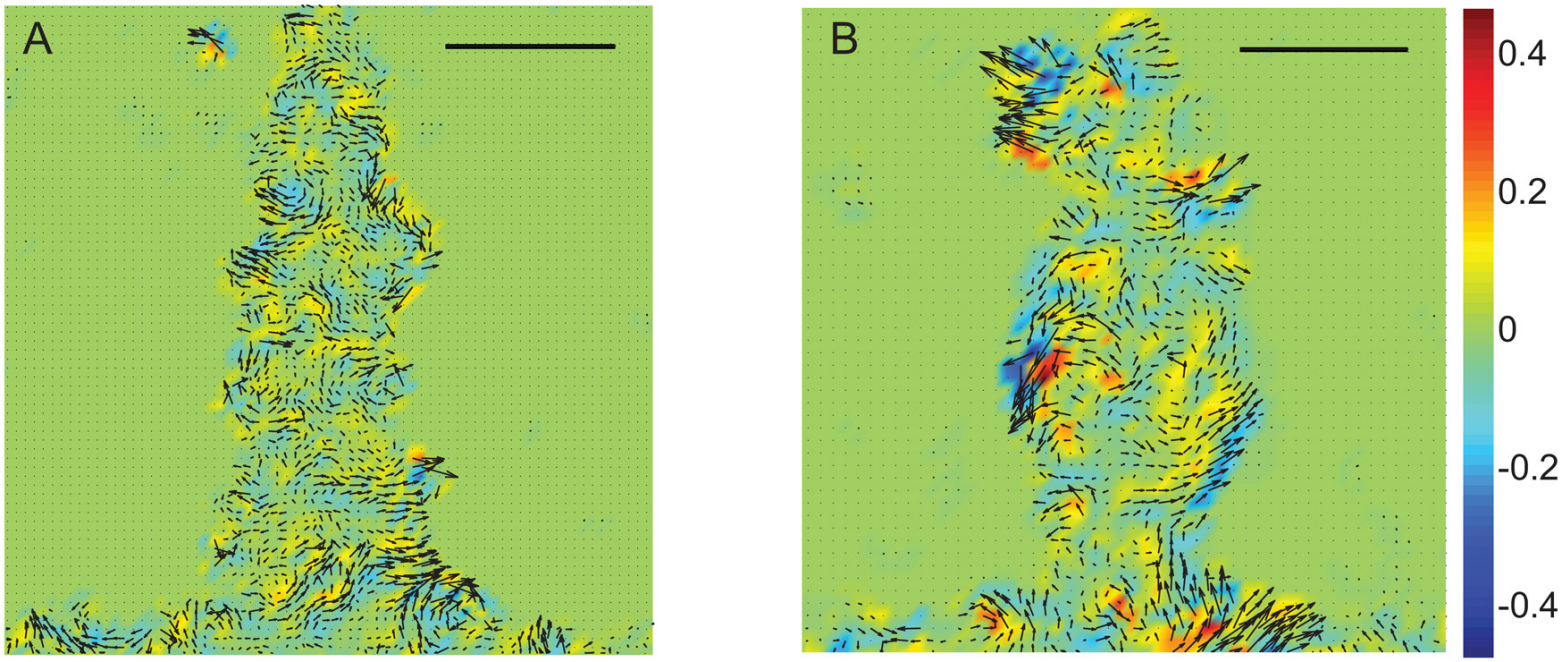
Velocity and vorticity plots are shown for cell clusters treated with 50 μM blebbistatin to inhibit myosin2 and alter cytoskeletal tension. A. MCF7 at 30 hours and B. MDCK at 16 hours. Scale bar = 200 μm.

**Fig 13:**
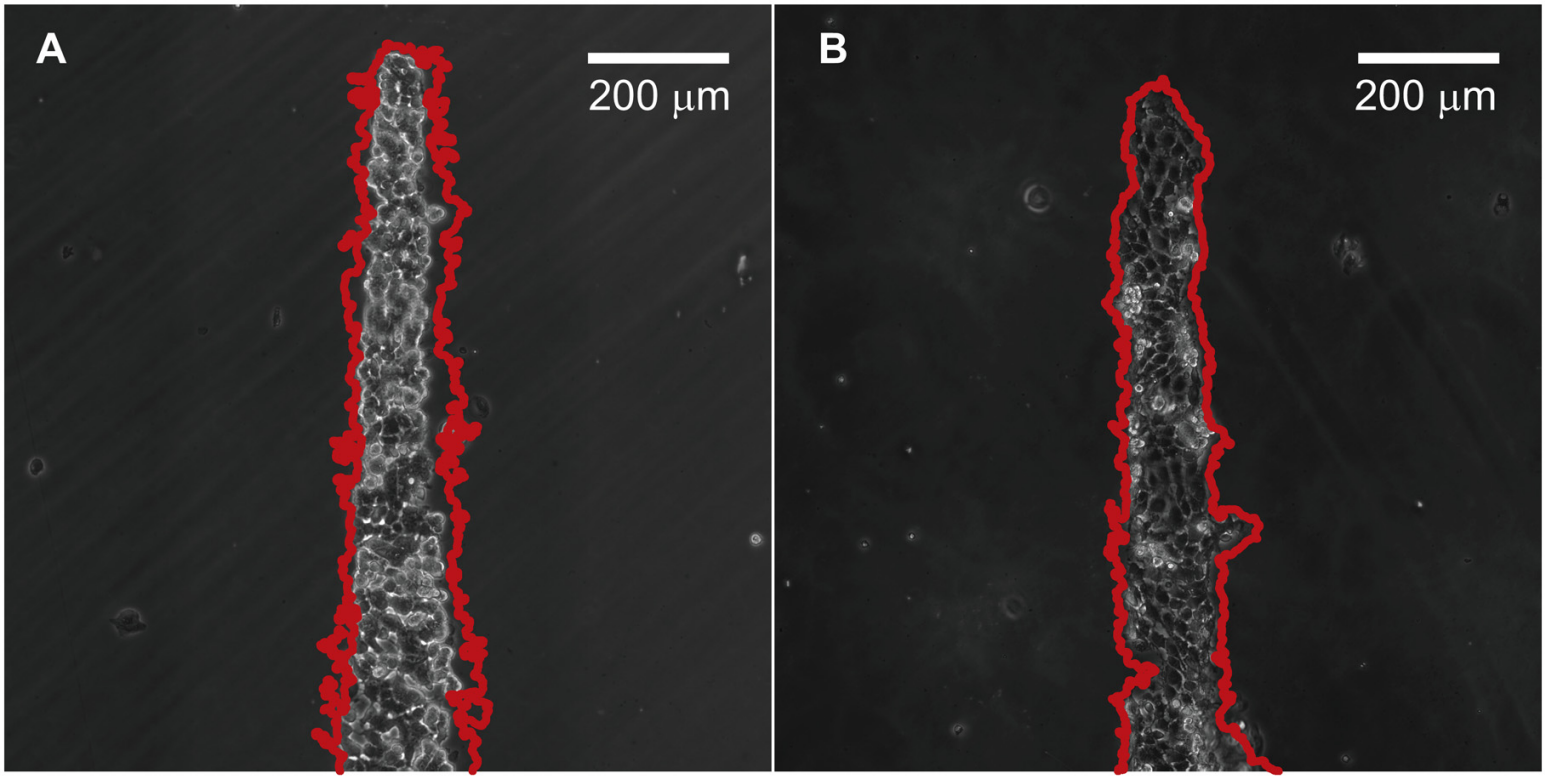
Effects of cytochalasin-D are shown for A. MCF7 cells and B. MDCK cells in cross shape at the start of the migration experiment. The edges of the cell ensembles are shown in red for MCF7 at 48 hours and MDCK at 16 hours which show minimal migrations over time.

Finger-like projections drag the front outwards which are important in collective migrations. The synergistic action of mechanical and chemical cues influence leader cell formation through differential spatial distribution of RhoA activity in the migration front [26]. Other chemical signalling molecules, such as Rac, integrin ß1, and P13K, localize specifically in leader cells and aid in driving collective migrations [33]. A variety of biochemical signals have been used to identify leader cells; the biophysical factors underlying their creation are however yet to be identified [34]. Stresses within individual cells and those between neighbouring cells are important in the collective migrations of cell monolayers. The transfer of local forces into global coordinated monolayer movements requires the integration of several factors including generation of forces, organization of actin within cells, and presence of E-cadherin to permit load transfer between cells. Acto-myosin stress fibers are, in addition, important in the transfer of loads within monolayers; their formation on the periphery of the ensembles is facilitated through mechanical stresses within the cells which are modulated through actin and myosin [20]. We conducted experiments to reduce the cytoskeletal tension using blebbistatin (50 μM) to inhibit myosin 2 and cytochalasin D (2 M) to depolymerize actin networks. MCF7 and MDCK cells, cultured in the cross shapes, were treated with blebbistatin (Fig 12) or cytochalasin-D (Fig 13) and the migration experiments were carried out to explore differences in the migration speeds as described earlier (S8 and S9 Videos).

**Fig 14:**
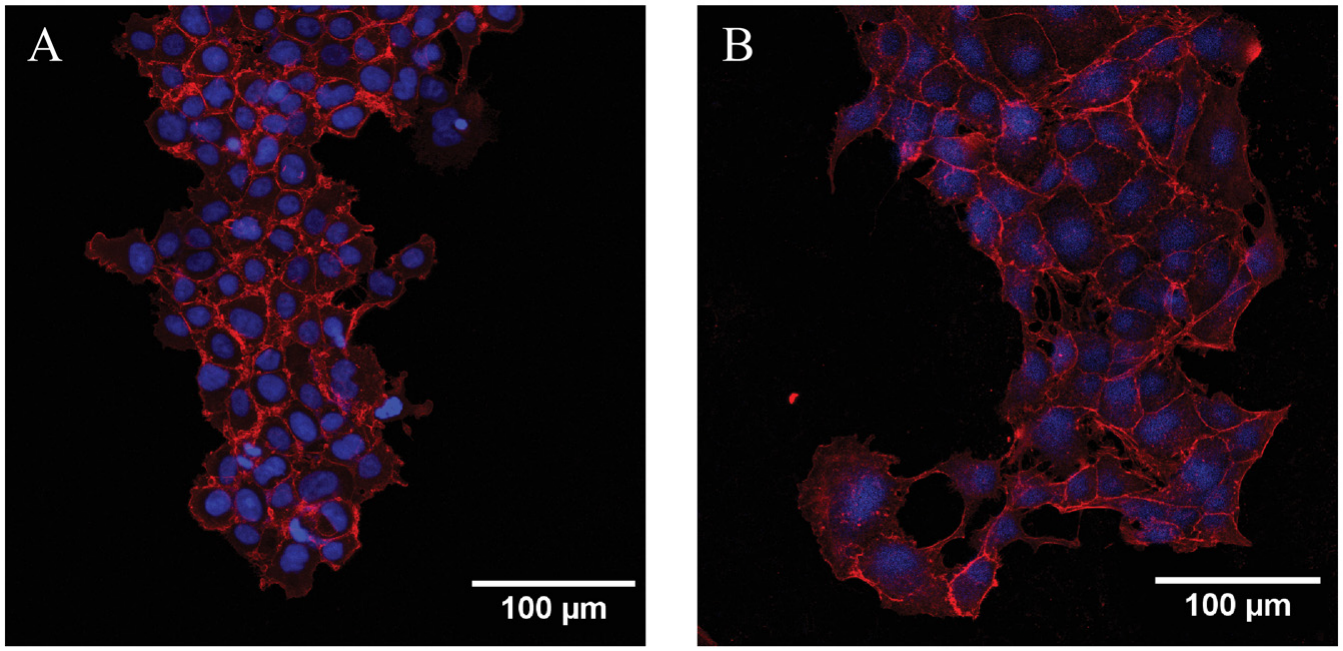
Fluorescence images of A. MCF7 cells at 48 hours and B. MDCK cells at 16 hours in the cross shape. There are few stress fibers seen in both cell types at the end of the experiments.

There are few differences, if any, in the migration speeds of the MCF7 and MDCK cell ensembles in the different regions of the cross shape due to the effects of blebbistatin; velocities in MDCK cells are however marginally higher than those in the MCF7 (Fig 12). There are few differences in the vorticities seen in MCF7 monolayers due to blebbistatin as compared to control untreated MCF7 ensembles (Fig 3). In contrast, MDCK cell monolayers treated with blebbistatin (Fig 13B) had significantly lower values of velocities and vorticities as compared to untreated MDCK cells (Fig 6). There are similar differences due to cytochalasin-D on the cell ensembles (Fig 13A and 13B) where we see minimal movement of the cells due to depolymerisation of actin. Corresponding fluorescence images of the individual cell clusters, treated with blebbistatin, in the cross are shown to visualize the presence of stress fibers (Fig 14). These results show the absence of the acto-myosin cables and organized stress fibers in the cluster monolayers for MCF7 and MDCK cells. The clear absence of acto-myosin cables bounding the MDCK cell ensembles and the corresponding lower values of vorticities in the blebbistatin treated MDCK cells as compared to control untreated cells shows the importance of the cable in the creation of leader cells and in the long range coordination of movements in cell ensembles. In an earlier study, Rausch and coworkers showed that a collection of epithelial cells may be polarized using sharp convex curvatures; the higher curvature regions correlated with a locally increased cellular motility and traction forces [20]. Leader cells, observed at highly curved protrusions and in regions within a curved perimeter, emerged from areas which had distinct openings in the actin fibers. We do not however see any breaks in the actin structures at regions of convex curvatures in our study that had a significantly lower migration speed at the tip regions of the cross shape. Together, these studies show the importance of actin fibers in the creation of leader cells from regions bounding the MDCK cell clusters. Mechanical constraining effects in producing coordinated movements in epithelial monolayers and their influence in inducing cell polarity presents an attractive approach to exploring the role of mechanics in cell signalling which are important in tissue engineering applications. The extent of influence of mechanical cues in directing cancer cell migrations and other processes, such as epithelial to mesenchymal transitions, however remains to be seen.

## Conclusions

Expanding cells in monolayers experience a variety of signals which include mechanical and chemosensory stimuli that synergistically contribute to collective migration behaviors of cells. Many of the biochemical regulatory mechanisms required for migration have been identified and mapped into comprehensive signalling pathways [18]. However, the underlying mechanical principles that guide cell clusters during migration are not yet fully understood. In this study, we investigate the effects of curvatures on migrations of initially constrained MCF7 and MDCK cell monolayers using methods in particle image velocimetry. Edges of the cell boundaries present asymmetric interfaces and have higher migration speeds as compared to cells in the interior. Cell monolayers expand, transfer forces to the interiors via cell-cell adhesions and pull the sheet outward. Three phases emerge temporally in the migrations of MDCK and MCF7 epithelial cells following removal of the constraint (Fig 5). Our results show that the migration speeds increase initially, following a plateau region of constant speeds and are accompanied with a decrease in the cluster speeds over longer time durations. Presence of an acto-myosin contractile ring in the MDCK cell clusters and the emergence of leader cells at regular intervals from the cross vertex show the importance of geometric curvatures to the overall migration behaviours of epithelial cell clusters. Our studies show vortices in the MDCK monolayers are clearly visible within 8 hours after release of the constraint in regions of interaction between the two arms of the cross. Cells in these regions are subjected to biaxial tension as compared to those in the cross arms and also have higher densities as compared to other regions of the cross. The role of substrate stiffness on the migration of epithelial cells has been demonstrated earlier but few studies have characterized the importance of geometric curvatures on cell monolayers. We also used cytoskeletal inhibitors to explore the individual roles of myosin 2 and actin in cell migrations. These studies show the importance of the bounding acto-myosin cables in producing leader cells and in the long range coordinated movements of cells. Biophysical parameters which influence the translation of local forces into global coordinated cellular migrations are yet to be identified. Our results suggest that geometric shapes and acto-myosin bounding cables in cell ensembles may polarize the cell and hence influence collective migrations. We hope these results are useful to tissue engineers to tailor surface roughness which influence cell migrations and to biophysicists who investigate the roles of mechanics in cancer cell signalling and migrations.

## Supporting Information

S1 Video Individual migrating cells (MCF7) were tracked using custom software and are highlighted using a red boundary with the centroid marked using red. Time between frames is 10 minutes.
(MP4)

S2 Video MCF7 cells migrate uniformly from circular constraints and are shown over 48 hours duration with 2 hours and 40 minutes between frames. (MP4)

S3 Video Cell migrations from a vertex of MCF7 cell clusters, initially constrained within a square shape, are shown for each frame at 2 hours and 40 minutes over a 48 hour period. The sharp vertex of the square smoothens rapidly with the onset of migration.
(MP4)

S4 Video Cell migration at the intersection of the arms of MCF7 cells constrained within cross shapes are shown over 48 hours as earlier. (MP4)

S5 Video MCF7 cells, initially constrained at the vertex of the cross shape, show minimal changes in their position during the migration experiment compared to cells located in the sides of the arm. (MP4)

S6 Video Collective cell migrations are shown at the intersection of MDCK cells initially constrained within cross shaped cluster for each 40 minute over a 16 hour duration. Cells move outward normal to the arm edges into cell-free regions until ~10 hours following which we see emergence of swirling vortices within the cell cluster.
(MP4)

S7 Video MDCK cells located at the cross vertices move significantly less as compared to those located in the arms of the cross. Leader cells are clearly seen to emerge at regular intervals in the sides of the arms by 3 hours.
(MP4)

S8 Video Blebbistatin treated MCF7 cells located in one arm of the cross are shown over the 48 hour duration of the experiment. Cells are less spread and migrate over shorter distances as compared to controls; the monolayer also does not maintain its integrity due to loss in the cytoskeletal tension.
(MP4)

S9 Video Corresponding collective cell migration in one arm of a blebbistatin treated MDCK cross shaped cluster over the 16 hours duration of the experiment. (MP4)
